# The functional roles of endophytic *Bacillus* and *Pseudomonas* bacteria to improve tomato and bean plants’ growth under induced-drought stress conditions

**DOI:** 10.3389/fpls.2026.1905932

**Published:** 2026-09-10

**Authors:** Laura Amaya-Quiroz, Mohammad Jamil Kaddoura, Mamta Rani, Jamil Samsatly, Hacene Meglouli, Saji George

**Affiliations:** 1 Department of Food Science and Agricultural Chemistry, McGill University, Montreal, QC, Canada; 2 BioSun Solutions, Chambly, QC, Canada

**Keywords:** abscisic acid pathway, bacterial endophytes, bioinoculants, crop productivity, drought stress, *Phaseolus vulgaris*, plant physiological response

## Abstract

Drought is a major constraint to crop productivity, highlighting the need for sustainable approaches to improve plant resilience. In this study, three endophytic bacteria, *Bacillus subtilis* BS-114 and BS-116, and *Pseudomonas wadenswilerensis* PPW-26, previously selected based on their plant growth-promoting traits and genomic potential, were evaluated for their ability to enhance drought resilience in common bean and tomato. Their effects were investigated through *in vitro* screening under polyethylene glycol (PEG)-induced drought, greenhouse drought validation, and open-field evaluation. Bacterial inoculation improved seed germination, seedling vigor, and plant water status under PEG-induced stress, with strain-dependent responses observed between crops, with the most pronounced effects observed in bean. Under greenhouse drought, inoculated bean plants exhibited improved photosynthetic performance and relative water content, together with reduced oxidative damage and proline accumulation, suggesting enhanced physiological adjustment to water deficit. These responses were associated with changes in the expression of drought-responsive genes involved in abscisic acid signaling, antioxidant defense, and osmoprotection, and were accompanied by a 52% increase in yield compared with non-inoculated drought-stressed plants. Based on greenhouse performance, a consortium containing BS-114 and two additional *Bacillus* strains was further evaluated under field conditions, where crop productivity was comparable to conventional NPK fertilization and a commercial algae-based biostimulant. Overall, these findings highlight the potential of beneficial endophytic bacteria isolated from medicinal plants to improve drought resilience, accompanied by changes in plant water status, hormonal signaling, and oxidative stress responses.

## 1 Introduction

Drought is a major environmental hazard that strongly affects agricultural productivity and food security. Global warming of ~1.1 °C above late-19th-century levels has intensified hydrological extremes such as floods and droughts. Many regions are experiencing more frequent and severe droughts and prolonged soil-moisture deficits, expanding areas under persistent water stress and increasing risks to crop yields and food security (Chiang et al., 2021; Intergovernmental Panel on Climate C, 2023). In Canada, drought is among the most costly and consequential climate-related hazards for agriculture, particularly in the Prairie provinces, where frequent and long-lasting droughts have repeatedly caused major economic losses and yield reductions. For instance, according to the Canadian Drought Monitor, as of May 2026, large areas of Canadian farmland were experiencing abnormally dry to severe drought conditions during the growing season (Agriculture and Agri-Food Canada, 2026). The Global Drought Outlook further estimates that the economic impact of an average drought event is currently up to six times higher than that in 2000, and drought-related costs are projected to increase by at least 35% by 2035 (OECD, 2025).

Reduced annual precipitation, depleted soil moisture, and lower surface and groundwater availability intensify abiotic stress in cropping systems and constrain plant growth and development (Dubey et al., 2021). Additionally, drought at various growth stages, such as vegetative growth and flowering, can seriously impair the performance of many crops, including leafy vegetables, cereals, legumes, fruits, and root crops (Dietz et al., 2021). Common beans (*Phaseolus vulgaris* L.), a warm-season legume crop, are particularly crucial for global food security as a source of iron, magnesium, and protein, especially in developing countries. However, bean cultivation regularly faces severe production challenges due to drought, with yield losses most pronounced during the reproductive period when water deficits disrupt flowering, pollen formation, and seed set (Geleta et al., 2024; Ponce et al., 2024). In previous studies on common bean under drought stress, oxidative damage increased, as indicated by elevated malondialdehyde (MDA) and hydrogen peroxide levels. Drought stress also disrupted plant water status, reduced photosynthetic pigments, and ultimately lowered grain yield compared with well-watered plants (Zamani et al., 2024).

Similarly, tomato *(Solanum lycopersicum)*, a globally important horticultural crop, is highly sensitive to drought stress. Drought reduces biomass accumulation and yield in tomatoes by disrupting growth, photosynthesis, and key physiological and metabolic processes (Ben Sedrine et al., 2024). A recent study on tomatoes reported that water stress reduced phytohormone levels such as indole acetic acid (IAA), gibberellic acid (GA), salicylic acid (SA), by up to 90%, and almost 50% decreases were observed in root and shoot biomass and leaf area. Drought also lowered chlorophyll content and leaf relative water content in tomato seedlings by 17% and 23%, respectively. These changes indicate impaired root function, limiting water and nutrient uptake and reducing photosynthetic capacity, ultimately constraining vegetative growth and reproductive performance (Turan et al., 2023). Overall, under drought, crops activate complex physiological responses that are regulated by drought-inducible genes and stress-signaling pathways, including mitogen-activated protein kinases, calcium-dependent protein kinases, carbohydrate partitioning pathways, and abscisic acid (ABA)-dependent signaling and ubiquitination pathways. The cumulative effects of these physiological and metabolic disturbances can reduce crop productivity by more than 40% under severe drought conditions compared with normal growing seasons, making agriculture the sector most affected by drought-related economic losses (Statistics Canada, 2022).

Increasing scientific evidence suggests that interactions with beneficial microorganisms, particularly plant growth-promoting bacteria and bacterial endophytes, can markedly enhance plant drought responses. Bacterial endophytes colonize internal plant tissues without causing disease and establish close associations with their hosts, allowing them to directly influence plant physiology and stress adaptation. As bioinoculants, these microorganisms have attracted considerable attention as sustainable alternatives to conventional agricultural inputs because they enhance plant growth and resilience through multiple complementary mechanisms. These include modulation of phytohormone activity (Figueiredo et al., 2008), promote the accumulation of organic osmolytes such as amino acids and sugars (Sandhya et al., 2010; Chen et al., 2017), produce ACC deaminase (Saikia et al., 2018) and diverse volatile organic compounds, and activate stress-responsive transcription factors (Paul et al., 2022) as demonstrated in several crops including beans, wheat, and maize.

Despite these promising findings, important knowledge gaps remain regarding the practical application of bacterial endophytes for enhancing crop resilience to drought. Most previous studies have focused on individual bacterial strains evaluated under controlled laboratory or greenhouse conditions and in a single crop species, leaving it unclear whether complementary bacterial consortium provide greater or more consistent benefits than individual inoculants. Furthermore, relatively few studies have examined whether endophytes selected through *in vitro* screening and greenhouse drought assays maintain their growth-promoting potential under open-field conditions. Moreover, the capacity of bacterial endophytes isolated from non-host medicinal plants to enhance drought tolerance in staple food crops, together with the physiological, biochemical, and molecular mechanisms underlying these cross-host beneficial interactions, remains poorly understood.

To address these knowledge gaps, this study investigated whether selected endophytic *Bacillus* and *Pseudomonas* strains, applied individually or as a consortium, can enhance drought resilience in common bean and tomato. Drought stress responses were evaluated under control *in vitro* and greenhouse conditions. Moreover, field trials were conducted under open-field conditions without imposing drought to assess plant responses and the effects of bacterial inoculation on crop performance. We hypothesized that selected endophytic strains would improve plant resilience to drought by modulating physiological, biochemical, and molecular responses, thereby contributing to improved crop performance under agricultural conditions.

## 2 Materials and methods

### 2.1 Bacterial strains used in this study

Based on the previously characterized genetic diversity of strains isolated from *Cannabis sativa* and *Chelidonium majus* (Amaya-Quiroz et al., 2026), five bacterial strains exhibiting plant growth-promoting traits and abiotic stress–related compound production were selected for this study. The selected strains included three *Bacillus subtilis* isolates, BS-114 (GenBank assembly GCA_054481215.1) and BS-116 (GenBank assembly GCA_054481175.1), BS-120 (GCA_054481115.1), one *B. mojavensis* strain BSM-119 (GCA_054481135.1), and one *Pseudomonas wadenswilerensis* PPW-26 (GenBank assembly GCA_054481295.1). Comprehensive morphological, biochemical, and genomic characterizations of these isolates were reported previously (Kaddoura et al., 2026).

### 2.2 Bacterial inoculum preparation

Bacterial strains were routinely maintained on Luria-Bertani (LB) agar and stored long-term in LB broth with 50% glycerol at −80 °C. For the inoculum preparation, single colonies from 24 h old cultures were transferred to LB broth for 24 h at 28 ± 1 °C in a rotary shaker (180 rpm). The cell suspensions were adjusted to an optical density (OD_600_) of 0.8, corresponding to approximately 10^8^ CFU·ml^-1^. Two bacterial inoculum preparations were used in this study depending on the experimental system: unfractionated bacterial suspension (UBS), consisting of bacterial cultures in their original LB medium without centrifugation, for *in vitro* assays; and bacterial cell suspension (BC), consisting of washed bacterial cells resuspended in sterile distilled water after centrifugation, for greenhouse and field experiments.

### 2.3 Estimation of exopolysaccharide content

Exopolysaccharide (EPS) production was quantified under increasing levels of osmotic stress conditions generated by supplementing growth media with 10% and 15% PEG-8000 as reported earlier (Ansari and I, 2018). The initial bacterial inoculum was adjusted to 10^8^ CFU·ml^-1^. Then, 1 mL of inoculum was transferred into 50 mL LB broth and incubated under the respective stress conditions at 28 °C for 5 days at 180 rpm. Cultures were centrifuged at 10,000 rpm for 20 min at 4 °C, and the supernatants were collected for EPS extraction. EPS was precipitated by adding cold absolute ethanol (1:2 v/v) and incubating at 4 °C overnight, then recovered by centrifugation (10,000 rpm, 10 min, 4 °C), dried at 55 °C overnight, and weighed to determine EPS yield.

### 2.4 Effect of bacterial inoculation on bean and tomato seedlings under PEG-drought stress

#### 2.4.1 Experimental design for germination stage

*In vitro* assays were conducted to assess the effects of bacterial treatments on seed germination of common beans (cv. Gold Rush Yellow) and tomatoes (cv. Beefsteak) under induced mild-drought stress (15% and 10% polyethylene glycol (PEG) 8000, respectively), following established protocols (Alwutayd et al., 2023; Du et al., 2025). Bean and tomato seeds were surface-sterilized in 2% NaOCl for 10 min and then incubated for 30 min in either distilled water (non-inoculated control; NC) or a log-phase UBS prepared as described in Section 2.2 (10^8^ CFU·mL^-^¹). Individual bacterial treatments consisted of BS-114, BS-116, or PPW-26, with each UBS diluted to 5% (v/v) in sterile distilled water before seed inoculation. Ten treated seeds were then placed on sterile filter paper in a Petri dish moistened with 15 mL of either sterile distilled water (well-watered control, (w+)) or 15% PEG 8000 (drought stress, (w-)). Similarly, for tomato, 12 treated seeds were kept in sterile petri-dishes containing half-strength MS medium supplemented with or without 10% PEG 8000. The experiment used three replicate plates per treatment, kept at 25 °C in the dark for 5 days. At the end of the experiment, germination percentage (Equation 1), root length, and seedling vigor index (SVI) (Equation 2) were determined.

(1) $$Germination\;percentage(\%)=\frac{number\;of\;germinated\;seeds}{total\;number\;of\;seeds}x\;100$$

(2) SVI=germination rate x root length at 5 DAS

#### 2.4.2 Experimental design for seedling stage

Protocol described by Alwutayd et al. (2023) (Alwutayd et al., 2023) was used for post-germination stress assays with some modifications. Five-day-old bean and tomato seedlings initially grown under well-watered conditions (w+) were transferred to 15% or 10% PEG 8000 media (w-), to induce drought conditions, and maintained in a growth chamber at 25 °C under a 16-h light/8-h dark cycle for 5 days. For bean seedlings, primary root length was recorded, and total seedling water content was calculated following Equation 3 at 5 days after stress (DAS). For tomato seedlings, root and shoot lengths were measured, and the root: shoot ratio was calculated.

(3) $$Water\;content\;(g\;H2O\;g^{-1}dry\;mass)=\frac{fresh\;mass-dry\;mass}{dry\;mass}$$

#### 2.4.3 Scanning electron microscopy imaging of tomato roots

Ten-day-old transversal sections of tomato roots (inoculated and non-inoculated) were fixed, dehydrated through an ethanol series, critical-point dried, sputter-coated, and examined by scanning electron microscopy FlexSEM (Hitachi High-Tech Corporation, Tokyo, Japan).

### 2.5 Evaluation of bacterial inoculation in beans under greenhouse drought conditions

#### 2.5.1 Plant growth experiment

Common beans were cultivated in a controlled environment at the McGill University MacDonald campus greenhouse facility (QC, Canada) to assess the impact of bacterial inoculation under drought stress. The experiment consisted of 64 pots (1-gallon, arranged on a 4.4 × 1.6 m bench), randomized with eight biological replicates per treatment. Seeds were primed with respective treatments before sowing, followed by four applications *via* soil drenching with 20 mL/pot. Drip irrigation was provided at 100 mL/day/pot. Treatments included: Non-inoculated control (NC), chemical control (NPK 18-18–21 at 0.5%), single-strain BC (BS-114, BS-116, or PPW-26, each applied at 5% v/v, prepared as described in Section 2.2), consortium (CONS: BS-114 + PPW-26 at 5%), CONS + NPK 50% dose (CONS.NPK_50,_ applied at 1:1 at 5%), and CONS + NPK at 100% dose (CONS.NPK_100_, applied at 1:1 at 5%). Parallel experiments under well-watered conditions were conducted for all treatments and served as controls (indicated by w+). Drought stress (w−) was induced by reducing irrigation to gradually decrease the relative soil moisture level from approximately 80% to 30%, as indicated by a portable digital soil moisture meter equipped with an electrical conductivity-based probe (Mcbazel, China). Soil moisture readings were monitored throughout the stress period, and irrigation was adjusted accordingly to maintain the target moisture levels. Plants were maintained under drought conditions for 15 days, followed by a 15-day recovery period under well-watered conditions. The experiment concluded at the first harvest, approximately 8–9 weeks after planting.

#### 2.5.2 Measurement of plant growth and stress-related parameters

Plant growth and yield metrics, including height, bud and flower number, were recorded throughout the experiment. Leaf samples were analyzed for relative water content (RWC) (Mukhtar et al., 2023), MDA (Siddiqui et al., 2015), and proline content (Shabnam et al., 2016) as indicators of water status and stress response. RWC included five biological replicates, and for MDA and proline, three biological replicates were used.

#### 2.5.3 Assessment of gas exchange indexes

Leaf-level gas exchange parameters, including photosynthetic rate (A) (µmol CO_2_ m^-2^ s^-1^), intercellular CO_2_ concentration (Ci) (µmol mol^-1^), stomatal conductance (Gsw) (mol H_2_O m^-2^ s^-1^), and transpiration rate (E) (mmol H_2_O m^-2^ s^-1^) were measured using LI-6800 portable photosynthesis system (LI-COR Biosciences, NE, USA). The third healthy lateral leaf counted from the tip of each plant was selected for measurements, and two data points within the same leaf were recorded for each measurement. The experiment was conducted with five plants (biological replicates) for each treatment. All determinations were conducted under ambient greenhouse conditions, in mid-morning, between 8:00–11:00 AM.

#### 2.5.4 Measurement of differential expression of selected genes by real-time PCR

##### 2.5.4.1 RNA extraction and cDNA synthesis

Total RNA was extracted from leaf tissues collected from three independent biological replicates per treatment using the RNeasy Plant Mini Kit (Cat. No. 74104, QIAGEN, Venlo, Germany) according to the manufacturer’s protocol. Genomic DNA contamination was removed by DNase I treatment (DNase I Kit, Promega, Madison, WI, USA). RNA quantity and integrity were assessed using a NanoDrop spectrophotometer (ND-2000, Thermo Fisher Scientific, Waltham, MA, USA) and by 1% agarose gel electrophoresis. cDNA was synthesized from 1 μg total RNA using the AffinityScript qPCR cDNA Synthesis Kit (Cat. No. 600559, Agilent Technologies, Santa Clara, CA, USA).

##### 2.5.4.2 Real-time polymerase chain reaction

Differential gene expression was analyzed by qRT-PCR using the Brilliant II Ultra-Fast SYBR^®^ Green QPCR Master Mix (Cat. No. 600882, Agilent Technologies, Santa Clara, CA, USA) on a CFx Maestro real-time PCR system (Bio-Rad, Hercules, CA, USA), with gene-specific primers detailed in **Supplementary Table 1**. The PCR cycling conditions included an initial denaturation at 95 °C for 3 minutes, followed by 40 cycles of denaturation at 95 °C for 5 seconds and combined annealing/extension at 55 °C for 10 seconds. Relative gene expression in control and drought-treated samples was normalized to *actin-11 (ACT11)* and computed using the 2^−ΔΔCt method (Livak and Schmittgen, 2001). Gene expression analysis was performed using three biological replicates per treatment, with each biological replicate analyzed in duplicate technical qPCR reactions. No-template controls were included in each qPCR run. The studied genes included *9-cis-Epoxycarotenoid Dioxygenase (NCED), Lipid Transfer Protein (LTP41), Early Responsive to Dehydration (ERD10), Late Embryogenesis Abundant protein (LEA) Heat Shock Protein DnaJ (DnaJ3), and Pyrroline-5-Carboxylate Synthase (P5CS).* These genes were selected as established drought-responsive markers encompassing signaling, osmotic adjustment, and cellular protection (Gutierrez-Benicio et al., 2016; López et al., 2020; Dong et al., 2022; Dong et al., 2022).

### 2.6 Effect of bacterial inoculation on bean and tomato productivity under open-field conditions

#### 2.6.1 Field studies in bean

Common bean was grown in soil at Lavaltrie, QC during June to August 2023 (daily temps: 19–21 °C; highs 26–28 °C, lows 14–16 °C; July +1–2 °C above normal; dew points 14–16 °C; RH 65–75%; 9–11 rainy days/month, 250–300 mm total precipitation, soil type-clay loam). Treatments included a chemical control (NPK 18-18-21, 50 mL at 0.5% w/v), an algae-based commercial biostimulant (CB, 50 ml at 0.6% v/v), and consortium (CONS: 50 mL total BC of BS-114, BSM-119, BS-120 at 5% v/v, 1:1:1 ratio). A randomized complete block design was implemented, consisting of four blocks per treatment, with a total of 80 plants per treatment. Treatments were applied four times during the growing season by soil drenching at the root zone. Pods were harvested three times in August 2023, mean yield (kg block^-1^) and mean yield per plant (kg plant^-1^) were reported.

#### 2.6.2 Field studies in tomato

Tomato was grown at Saint Rémi, QC during June to September 2025 (daily temps: 19–22 °C; highs 26–28 °C, lows 14–16 °C; dew points 14–17 °C; RH 65–75%; 9–12 rainy days/month, soil type-clay loam). June heat reached 34.5 °C (dew point 25.4 °C). Treatments included a chemical control (NPK 18-18-21, 50 mL at 0.5% w/v), an algae-based commercial biostimulant (CB, 50 ml at 0.6% v/v), and consortium (CONS: 50 mL total BC of BS-114, BSM-119, BS-120 at 5% v/v, 1:1:1 ratio). The experiment was conducted as a single-plot (non-replicated) preliminary study due to limited field availability. Each treatment was assigned to one contiguous field line containing approximately 90 plants, with the treatment line considered the experimental unit. Treatments received four drench applications during the growing season. Total yield was determined by harvesting all plants within each treatment line across three late-season harvests (ending September 10 and 29) and was expressed as kg line^-1^. Mean yield per plant (kg plant^-1^) was calculated from total line yield divided by the number of plants established per treatment line.

### 2.7 Statistical analysis

Data processing and statistical analyses were performed using Microsoft Excel and GraphPad Prism (v10.6.1) (GraphPad Software, CA, USA). A two-way analysis of variance (ANOVA) was used for experiments evaluating the effects of bacterial treatment, water regime (well-watered vs. drought), and their interaction, including germination percentage, SVI, and proline and MDA content. A one-way ANOVA was used for experiments involving a single experimental factor, including root length, seedling water content, root-to-shoot ratio, bacterial EPS production, and all greenhouse growth and physiological measurements. When significant differences were detected, Tukey’s honestly significant difference test was used for pairwise comparisons, whereas Dunnett’s test was applied when comparing bacterial treatments with the water-treated control. Results are presented as mean ± standard deviation, and statistical significance was accepted at p < 0.05. All experiments were conducted with a minimum of three biological replicates unless otherwise specified.

## 3 Results

### 3.1 Effect of bacterial inoculation on bean and tomato seedlings under PEG-drought stress

**Figure 1:**
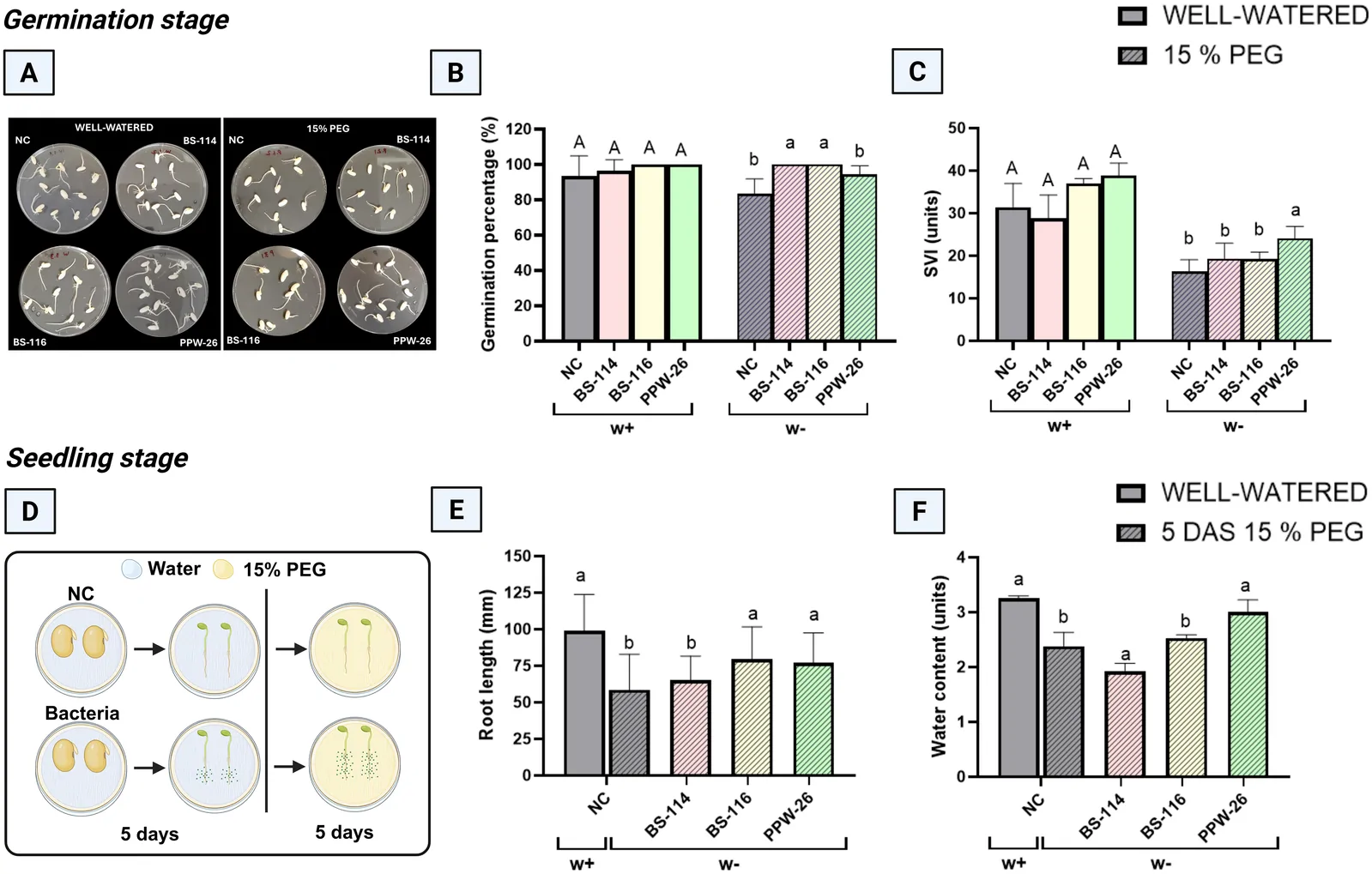
Effects of microbial inoculation on bean seedling traits under well-watered and drought conditions. **(A)** Representative seedlings, **(B)** germination percentage, **(C)** SVI at 5 days. Data are means ± SD (n = 3). Different letters denote treatments that differ significantly from the NC (two-way ANOVA followed by Dunnett’s test, P < 0.05). **(D)** Schematic overview of the seedling experiment timeline. **(E)** Root length and **(F)** seedling water content at 10 days (5 DAS). Data are means ± SD, n = 30, n = 3, respectively. Different letters denote significant differences from the NC (one-way ANOVA followed by Dunnett’s test, P < 0.05). NC, non-inoculated control; SVI, seedling vigor index.

Under drought stress, bean seed germination in NC(w−) decreased by 10% compared with NC(w+) (**Figures 1A, B**). Inoculation with BS-114(w−) and BS-116(w−) significantly increased germination by 16.7% relative to NC(w−). PPW-26(w−) treatment showed a similar trend; however, did not differ significantly from the drought control. The detrimental effect of drought was also reflected in SVI, which decreased significantly by 48% in the NC(w-) relative to NC(w+) (**Figure 1C**). Compared with NC(w−), PPW-26(w−) significantly increased SVI by 48%. Similarly, improvements were observed in BS-114(w−)- and BS-116(w−)-inoculated seedlings (**Figure 1D**). At the seedling stage, 15% PEG decreased both root length and whole-seedling water content (**Figures 1E, F**). Inoculation with BS-116(w-) and PPW-26(w-) significantly enhanced root growth relative to NC(w-). Water content results showed that PPW-26 significantly increased seedling water content to levels comparable to NC(w+).

**Figure 2:**
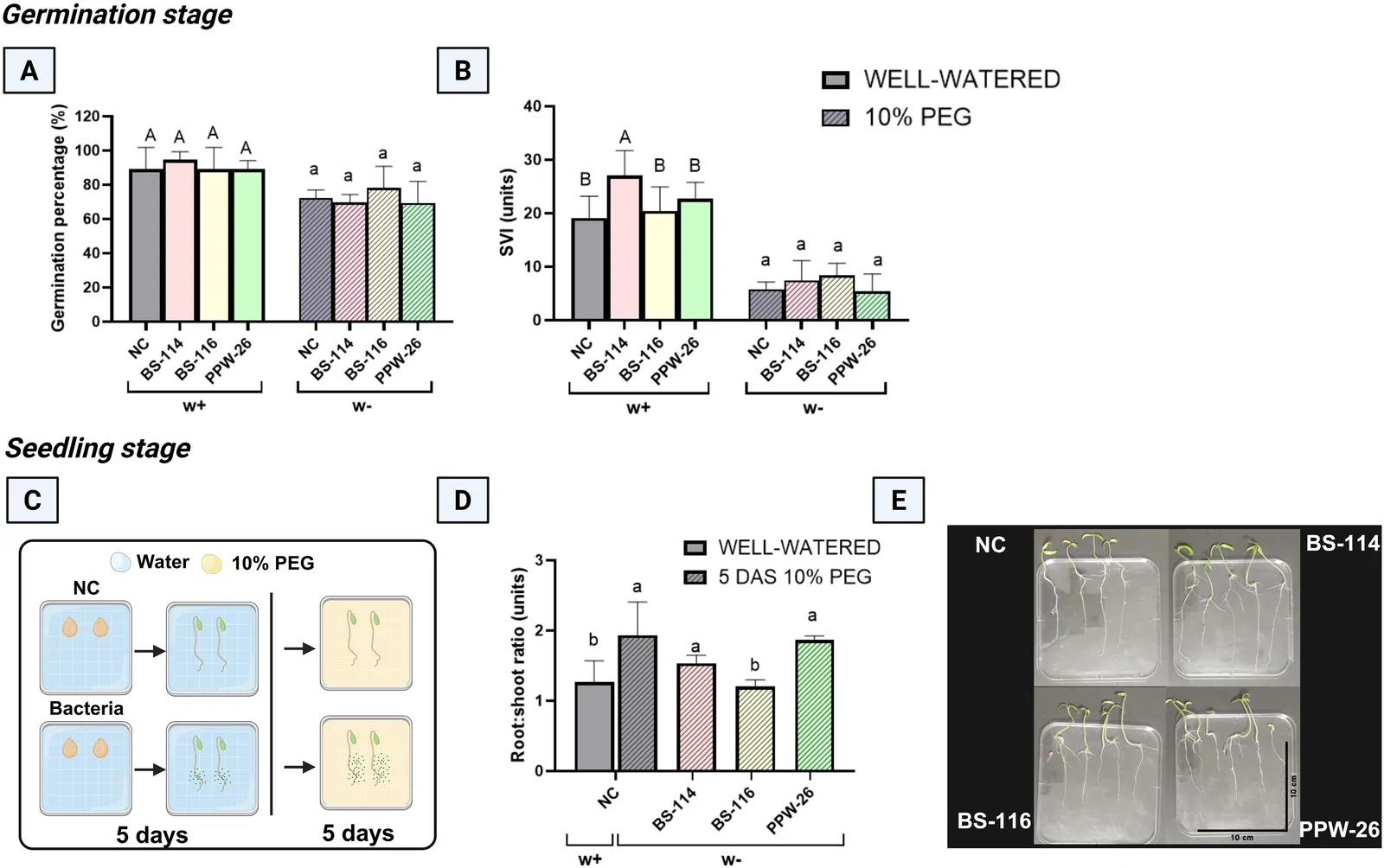
Effects of microbial inoculation on tomato seedling traits under well-watered and drought conditions. **(A)** Germination percentage **(B)** SVI at 5 days. Data are means ± SD (n = 3). Different letters denote treatments that differ significantly from NC (two-way ANOVA followed by Dunnett’s test, P < 0.05). **(C)** Schematic overview of the seedling experiment timeline. **(D)** Root/shoot ratio of tomato seedlings at 10 days (5 DAS). Data are means ± SD, n = 36. Different letters denote significant differences from NC (one-way ANOVA followed by Dunnett’s test, P < 0.05). **(E)** Representative seedling at 5 DAS. NC, non-inoculated control; SVI, seedling vigor index.

For tomato, 10% PEG reduced the seed germination rate by 16.7% in NC(w-) in comparison to NC(w+). Only BS-116(w−) showed a measurable increase compared with NC(w−); however, this difference was not statistically significant (**Figure 2A**). Moreover, NC(w+) showed an SVI of 22.3, which significantly decreased to 6.7 in NC(w-). Although BS-114(w−) and BS-116(w−) showed numerical increases in SVI compared with NC(w−), these differences did not reach statistical significance (**Figure 2B**). For biomass allocation, both the NC(w+) and BS-116(w-) treatments showed a lower root:shoot ratio in comparison to NC(w-). This pattern indicates that BS-116 inoculated seedlings partly shifted biomass back towards shoots, similar to NC(w+) (**Figures 2C–E**).

Collectively, these *in-vitro* assays revealed a more pronounced bacterial-mediated improvement in drought tolerance traits in bean compared with tomato. Therefore, BS-114, BS-116, and PPW-26 were selected for greenhouse evaluation using bean as the plant model to further assess their effectiveness under soil-based growing conditions.

### 3.2 Evaluation of bacterial inoculation in beans under greenhouse drought conditions

#### 3.2.1 Growth and physiological parameters

**Figure 3:**
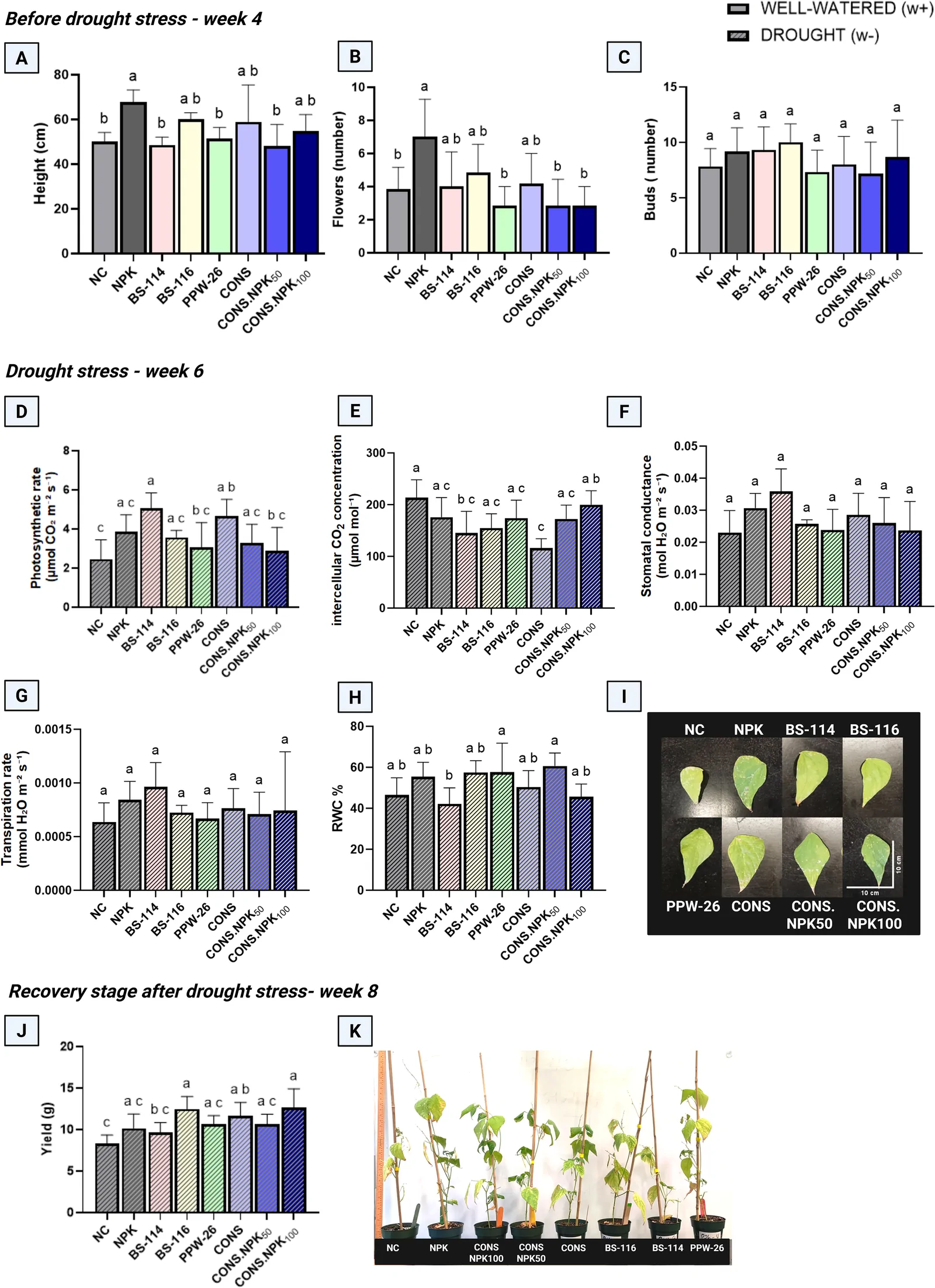
Growth and physiological parameters at different stages. **(A)** Plant height, **(B)** number of flowers, and **(C)** number of buds. Data are means ± SD (n=6). **(D)** Photosynthetic rate, **(E)** intercellular CO_2_ concentration, **(F)** stomatal conductance, **(G)** transpiration rate, and **(H)** RWC. Values are means ± SD (n = 5). **(I)** Representative leaf appearance under different treatments. **(J)** Average yield of per plant. Data are presented as means ± SD (n=5). **(K)** Representative bean plant at the end of the experiment for each treatment. Different letters indicate significant differences among treatments (one-way ANOVA followed by Tukey’s test, P < 0.05).

At the initial stage of the experiment, prior to drought stress imposition, BS-116 and CONS increased plant height by 20.2% and 17.6%, respectively, relative to NC (**Figure 3A**). Notably, these treatments did not differ significantly from the NPK treatment. Similarly, BS-116, CONS, and BS-114 increased flower number by 26.3%, 10.5%, and 5.3%, respectively, compared with NC, indicating a moderate positive effect on reproduction, while again showing no significant difference from NPK (**Figure 3B**). BS-116 and BS-114 increased bud number by 28.2% and 19.2% over NC, respectively (**Figure 3C**). Overall, BS-116, and to a lesser extent CONS and BS-114, improved height and especially reproductive traits relative to NC, with values comparable to NPK treatments.

Physiological traits such as photosynthetic rate (A), intercellular CO_2_ concentration (Ci), stomatal conductance (Gsw), and transpiration rate (E) in the group NC(w-) were reduced after two weeks of drought stress relative to NC(w+) reference values reported in a parallel study from our group (Kaddoura et al., 2026), with decreases of 51.5%, 34.7%, 75.3%, and 52.4%, respectively. Under drought, A and Ci showed significant differences. BS-114(w-) and CONS(w-) significantly increased A relative to NC(w-) (by 106% and 89.4%) (**Figure 3D**), while showing the lowest values of Ci (**Figure 3E**). Although Gsw and E were not statistically different among treatments, BS-114(w-) upregulated Gsw and E by 55.5% and 51.4% relative to NC(w-) (**Figures 3F, G**). Moreover, RWC decreased from 67.6% in NC(w+) to 46.6% in NC(w-) (**Supplementary Figure 1**). Notably, RWC increased by 30.3%, 23.8%, and 23.4% in CONS.NPK50(w-), PPW-26(w-), and BS-116(w-), respectively, compared with NC(w-) (**Figures 3H, I**).

After two weeks of drought and two weeks of recovery, CONS.NPK100(w-), BS-116(w-), CONS(w-) produced significantly higher yields under drought, increasing yield by 52%, 50% and 40% relative to NC(w-) (**Figures 3J, K**).

#### 3.2.2 EPS production of inoculated strains and biochemical properties of leaves

**Figure 4:**
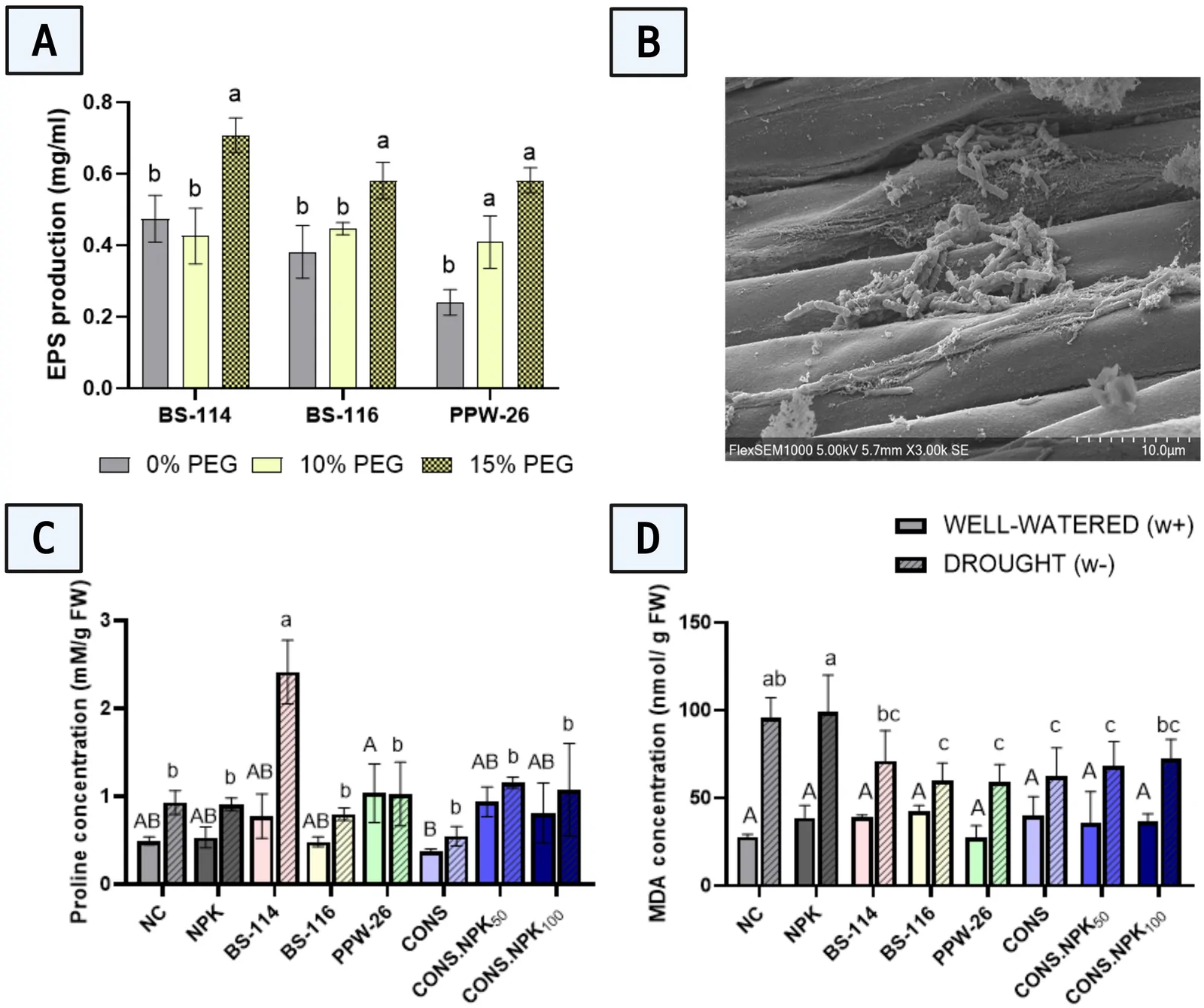
**(A)** EPS production by the strains used in this experiment under different PEG concentrations. Data are presented as means ± SD (n=3). Different letters indicate treatments that differ significantly from the non-inoculated control (one-way ANOVA followed by Dunnett’s test, P < 0.05). **(B)** SEM image of roots from bean seedlings inoculated with BS-116 **(C)** Proline concentration, and **(D)** MDA content in beans after recovery. Data are presented as means ± SD (n=3). Different letters indicate treatments that differ significantly from each other (two-way ANOVA followed by Tukey’s test, P < 0.05).

Bacterial EPS production and compounds associated with plant drought responses were evaluated. EPS production increased in all three strains as PEG concentration rose. The highest levels occurred at 15% PEG, where BS-114 produced 49% more EPS than under PEG-free conditions, and BS-116 and PPW-26 increased EPS by 52.5% and 141.9%, respectively (**Figure 4A**). Along with this higher EPS production, SEM imaging provided qualitative evidence of bacterial attachment to the root surface in BS-116-treated plants (**Figure 4B**).

After a two-week recovery period following drought stress, proline concentration and MDA content in bean leaves remained influenced by the previous drought treatment. Mean proline content increased from 0.68 in the NC(w+) to 1.11 units in the NC(w-), indicating osmotic stress. Under drought, CONS(w-) and BS-116(w-) reduced proline by 41% and 14.1% relative to NC(w-), suggesting lower stress in these plants (**Figure 4C**). MDA content, an indicator of lipid peroxidation, also increased under drought conditions, rising from 36.0 in NC(w+) to 73.6 in NC(w−), indicating enhanced oxidative stress. Under drought, PPW-26(w-), BS-116(w-), and CONS(w-) reduced MDA by 38.2%, 37.4%, and 34.4%, respectively, compared with NC(w-) (**Figure 4D**).

#### 3.2.3 Analysis of genes expression in response to drought

**Figure 5:**
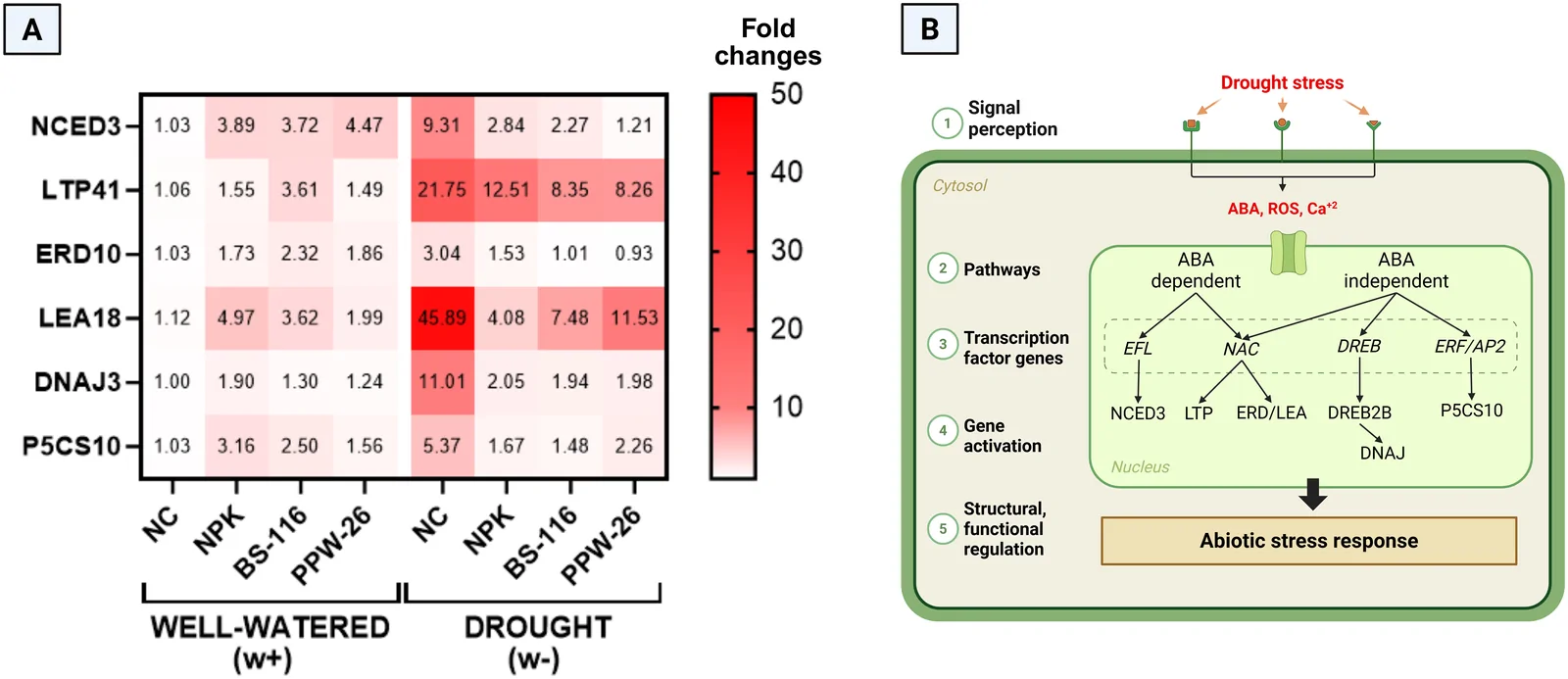
**(A)** Heat map showing the effects of bacterial treatments on the relative expression of drought-responsive genes in bean leaves. Expression values were calculated using the $2^{-\text{ΔΔ}Ct}$ method and normalized to the non-inoculated well-watered control [NC(w+)]. Red indicates increased transcript accumulation, whereas white represents values near the baseline (1-fold change). Data represents three biological replicates obtained from three independent plants (n = 3). **(B)** Schematic representation of model mechanism of bacterial-mediated drought tolerance in beans.

To gain insight into the effects of bacterial treatments following drought stress and subsequent recovery, expression levels of a panel of target genes were assessed by qRT-PCR. Heat maps of transcript expression were generated and data presented as relative expression changes with respect to the NC(w+) in **Figure 5A**. These targets represent key components of ABA-dependent and ABA-independent pathways, including osmolyte accumulation and cellular protection mechanisms (**Figure 5B**). In NC plants, the expression of all target genes was upregulated under drought stress compared with well-watered conditions.

NCED3 was strongly induced by drought in NC, with a 9.31-fold increase, but its expression decreased in plants treated with PPW-26 (1.21-fold) and BS-116 (2.27-fold). LTP41 and LEA18 were markedly upregulated under drought in NC (21.75- and 45.89-fold, respectively), whereas their expression was significantly reduced in plants treated with BS-116 and PPW-26. Similarly, ERD10 showed a 3.04-fold increase in drought-stressed NC plants, but only 1.01 and 0.93 fold in PPW-26 and BS-116 treated plants, respectively. DNAJ3 was also strongly induced in drought-stressed NC plants (11.01-fold) but downregulated in bacteria-treated plants. Finally, P5CS10 followed the same pattern, with upregulation in NC (5.37-fold) and downregulation in BS-116- and PPW-26-treated plants (**Figure 5A**).

### 3.3 Effect of bacterial inoculation on bean and tomato productivity under open-field conditions

Based on the greenhouse results obtained in this study and the plant growth-promoting traits previously identified during strain screening, BS-114 was combined with two additional Bacillus strains (BSM-119 and BS-120) (Kaddoura et al., 2026) to form a consortium treatment. The consortium was subsequently evaluated under open-field conditions to determine whether the plant growth-promoting effects observed under controlled drought experiments could be translated into consistent improvements in crop performance under non-controlled agricultural conditions.

**Figure 6:**
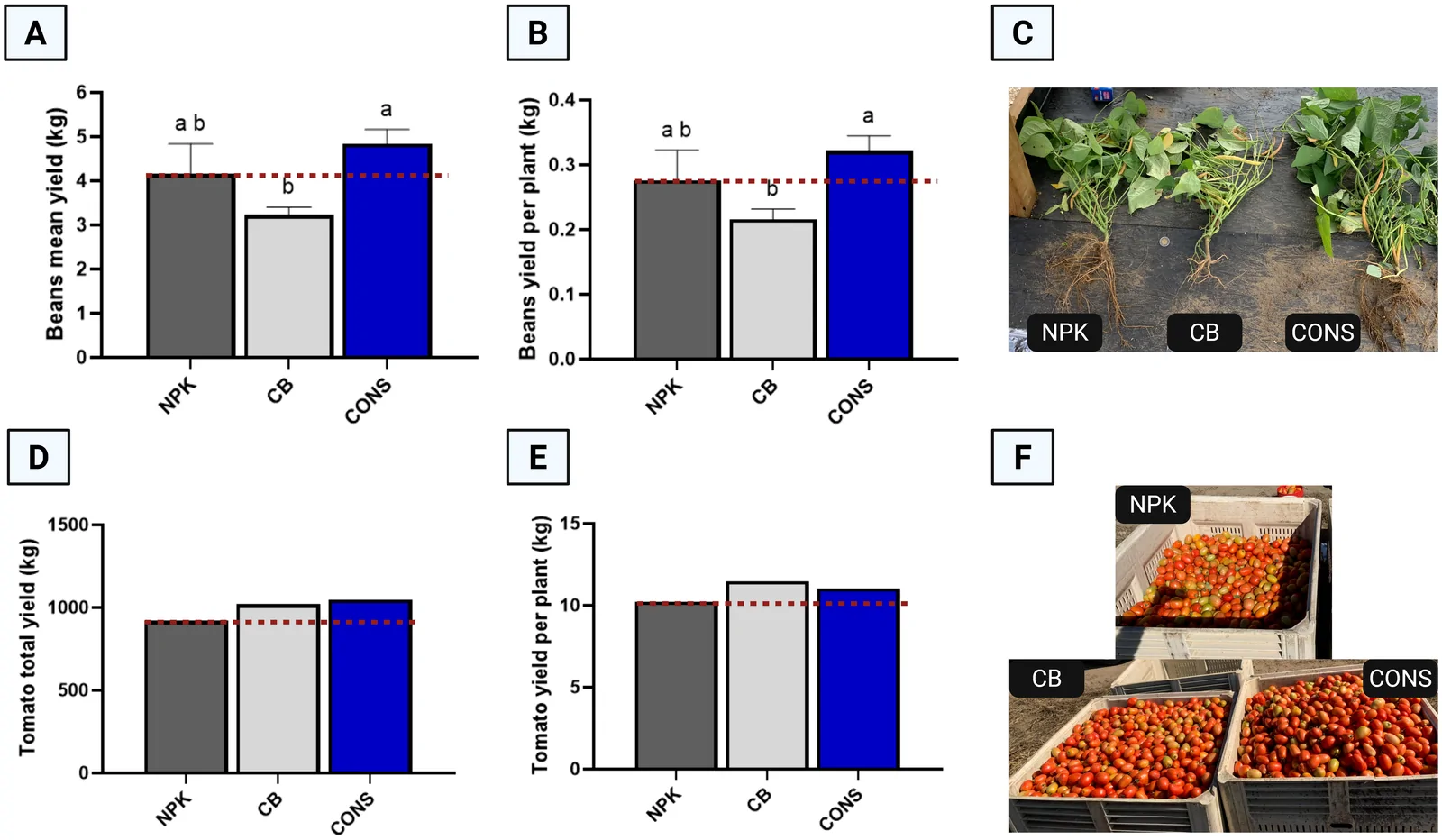
Summary of crop yield: **(A)** Total yield and **(B)** per plant in 2023. Data are presented as means ± SD (n=3). Different letters indicate significant differences among treatments (one-way ANOVA followed by Tukey’s test, P < 0.05). **(C)** Representative plants per treatment at harvest time **(D)** Total yield and **(E)** per plant in 2025 (n=1) **(F)** Representative harvested fruits per treatment. Treatments, from left to right, are: NPK; chemical control under farmer practice conditions; CB (commercial biostimulants); and CONS: BS-114, BSM-119, and BS-120.

In the bean field experiment, yield differed significantly among treatments. The CONS treatment produced the highest mean yield per block (4.84 kg), followed by NPK (4.16 kg), whereas CB resulted in the lowest yield (3.24 kg) (**Figure 6A**). Mean yield per block under CONS was significantly greater than that of CB (49.4% higher vs. CB), while no significant difference was detected between CONS and NPK. A similar trend was observed for mean yield per plant, with CONS producing the highest value (0.323 kg plant^-^¹) (**Figures 6B, C**).

The tomato field trial was conducted as a single-plot, non-replicated preliminary evaluation; therefore, the results are presented descriptively. The CONS treatment produced the highest total yield (1048.7 kg line^-^¹), followed by CB (1022.4 kg line^-^¹) and NPK (921.5 kg line^-^¹) (**Figure 6D**). A similar ranking was observed for mean yield per plant, with the highest values recorded for CONS, followed by CB and NPK (**Figures 6E, F**).

## 4 Discussion

Drought limits crop establishment and yield, especially during germination and early vegetative growth. Here, we tested the hypothesis that bacterial endophytes possessing plant growth-promoting traits can enhance plant performance and contribute to improved responses to abiotic stress in non-host plants. This study builds on previous work characterizing endophytic strains with biostimulant activity. These traits include indole production, siderophore and organic acid production, phosphate and zinc solubilization, biofilm formation, and proline and SOD accumulation (Kaddoura et al., 2026). Common bean was selected because its distinct vegetative and reproductive stages allow evaluation of drought effects on growth and yield, while its association with diverse rhizosphere microbial communities provides a relevant system for studying bacterial bioinoculation (Rocha et al., 2023). Tomato has been reported to show drought responses early stages of plant growth, including shifts in root-to-shoot allocation and root system architecture under water deficit, making it a suitable model for evaluating plant responses to drought stress (Korwin Krukowski et al., 2020).

Seed germination depends on environmental conditions and phytohormone balance, hence drought consistently reduces germination and seedling vigor across crops (Wu et al., 2021). In this study, bacterial inoculation improved several drought-related seedling traits under PEG-induced stress in both bean and tomato, although responses varied depending on the host–strain combination. The *Pseudomonas* isolate produced the greatest improvements in bean seedling vigor, root length, and tissue water content, whereas *Bacillus* strains mainly influenced biomass allocation in tomato by reducing the root ratio under drought. These responses are consistent with the plant growth-promoting traits previously characterized in the selected strains, particularly IAA and EPS production (Kaddoura et al., 2026). IAA likely promoted root development and early seedling growth, while EPS may have contributed to improved water retention around the root surface and supported bacterial persistence under osmotic stress (Niu et al., 2018). Consistent with this interpretation, previous work reported that EPS and IAA-producing bacteria improved soybean seed germination under 4% PEG-induced drought and enhanced germination and vigor of other crops under osmotic stress (Igiehon et al., 2019).

Changes in root:shoot allocation are commonly associated with drought adaptation because plants adjust carbon investment between below- and above-ground tissues to optimize water acquisition and carbon gain (Whitmore and Whalley, 2009; Ayaz et al., 2015). In this study, BS-116 reduced the root:shoot ratio under PEG stress, suggesting relatively greater investment in shoot biomass rather than root elongation. Our observations align with Arkhipova et al. (2007), who found that inoculating lettuce seedlings with a *Bacillus* strain decreased the root:shoot ratio by enhancing shoot growth and limiting root elongation (Arkhipova et al., 2007). Similarly, a recent study reported that moderate and severe water deficits reduced soil microbial activity and maize growth, decreasing shoot biomass and increasing the root:shoot ratio (Pereira et al., 2020).

A limitation of this *in vitro* screening approach is that seeds were inoculated with overnight bacterial cultures containing residual LB medium rather than purified bacterial cells. Although bacterial cultures were harvested after reaching the stationary growth phase, the contribution of residual medium components cannot be completely excluded. To eliminate this potential confounding factor, all subsequent greenhouse and field experiments were performed using bacterial cells washed and resuspended exclusively in sterile water before inoculation.

Overall, responses were stronger in bean than tomato, with each bacterial strain improving different seedling traits, suggesting strain-specific and potentially complementary mechanisms of action. Therefore, the three strains were advanced to greenhouse evaluation in bean, where they were tested individually and as a consortium to determine whether strain-specific benefits observed during early seedling establishment translated into improved whole-plant performance under controlled drought conditions. Bean also provided a suitable system to further investigate these plant–microbe interactions by evaluating bacterial performance within a developing rhizosphere microbiota rather than the simplified environment of the *in vitro* assay. Although the selected strains were characterized as EPS producers, EPS production and bacterial colonization on seedling roots were not quantified in this study. Qualitative SEM observations confirmed bacterial attachment to the root surface; however, future studies using fluorescently tagged strains combined with quantitative colonization assays will be required to directly link root colonization and *in situ* EPS production with the growth-promoting responses observed during early seedling establishment.

Experiments with the selected single strains and consortium were subsequently conducted under well-watered and drought conditions in greenhouse pot trials to evaluate their potential synergistic effects. Bacterial inoculation, which produces multiple plant growth–promoting and abiotic stress–related compounds, mitigated drought-induced impairments at morphological, physiological, biochemical, and gene expression levels, consistent with previous reports (Vishnupradeep et al., 2022; Choghakaboudi et al., 2025). Bioinoculation enhanced vegetative growth and reproductive development, as reflected by increases in plant height and flower number. These effects are commonly associated with the production of phytohormones such as IAA, which regulate cell division and elongation in roots and shoots, thereby improving water and nutrient uptake (Wu et al., 2016). Organic acid production may additionally contribute to nutrient mobilization under soil conditions, although this mechanism was not directly evaluated here (Chen et al., 2019). Previous study (Kaddoura et al., 2026) showed that the selected strains produce high levels of total indoles, siderophores, and organic acids, suggesting their growth-promoting effect. Moreover, this early stimulation of growth provides a basis for improved performance when drought occurs.

Drought stress significantly reduced gas exchange parameters in NC, limiting photosynthetic capacity mainly through decreased stomatal conductance and associated metabolic constraints. Bacterial inoculation increased CO_2_ assimilation while lowering Ci. However, stomatal conductance and transpiration remained similar to NC, indicating that the improvement in photosynthesis was not associated with increased stomatal opening. Instead, the reduction in Ci together with increased CO_2_ assimilation suggests enhanced CO_2_ utilization and improved biochemical carbon fixation capacity within the leaf. Although specific enzymatic processes were not directly measured, these responses are consistent with improved photosynthetic efficiency and maintenance of carbon fixation under drought conditions (Cornic and Ghashghaie, 1991). Similar improvements in gas exchange and recovery have been reported in drought-stressed sugarcane inoculated with *Bacillus subtilis* and in grapevine treated with *Acinetobacter* and *Pseudomonas* spp (Rolli et al., 2015; Fonseca et al., 2022).

Consistent with the reduction in photosynthetic performance, drought also decreased RWC in bean, whereas bioinoculation improved plant water status. Although not directly measured in the rhizosphere, EPS production may have contributed to root–soil interactions under water deficit (Naseem and Bano, 2014; Kim et al., 2024). This interpretation is supported by enhanced bacterial EPS production under drought, particularly by PPW-26, which increased RWC only under drought conditions, suggesting a stress-responsive effect. Other bacterial traits, including IAA and siderophore production, may have also promoted root development and nutrient acquisition. Together, these responses likely contributed to maintaining leaf hydration and photosynthetic function under drought (Batool et al., 2020).

Despite the general improvement in physiological traits, strain-specific responses were evident. BS-114 markedly increased photosynthetic rate but did not fully restore leaf RWC, supporting a predominant effect on photosynthetic activation rather than water status. This pattern suggests improved pigment stability and/or antioxidant protection that maintains photosynthetic machinery and carbon fixation under drought. Several studies show that bacterial inoculation can enhance CO_2_ assimilation, protect photosystems, and reinforce antioxidant defenses, thereby sustaining reproductive development and grain filling under water deficit (Naveed et al., 2014; Al-Amri, 2021). By contrast, BS-116 and PPW-26 mainly increased leaf RWC, indicating improved water retention and osmotic adjustment, while their effect on photosynthetic recovery remained modest. These strains appear to primarily improve tissue hydration and overall physiological stability, with yield gains associated with better flower and pod retention. These contrasting patterns indicate that *Bacillus* and *Pseudomonas* strains may be conferring drought tolerance through distinct modes of action.

Such functional diversity helps explain differences in yield responses and suggests that combining complementary strains could further enhance productivity under drought. This was supported by CONS, which showed increases in carbon fixation together with improved RWC, suggesting simultaneous enhancement of photosynthesis and water status. Similar results have been reported where bacterial consortium outperform single strains in maintaining gas exchange, RWC, antioxidant balance, and yield under drought (Saleem et al., 2021). This combined physiological improvement, likely contributed to the higher productivity observed.

MDA, a marker of membrane damage caused by drought-induced reactive oxygen species, remains a reliable indicator of cellular injury (Yang et al., 2021). Proline accumulation is also a key biochemical response to drought stress, contributing to osmotic adjustment, stabilization of proteins and membranes, and maintenance of cellular water balance (Arteaga et al., 2020). Beans inoculated with BS-116 showed the lowest MDA levels after recovery, suggesting that this high SOD-producing *B. subtilis* strain effectively scavenged ROS under drought stress. Enhanced ROS detoxification can reduce oxidative damage and lessen the need for osmotic protection, resulting in lower proline accumulation. Similar decreases in MDA and proline following microbial inoculation have been reported in maize, soybean, and wheat under drought conditions, where they were interpreted as indicators of improved stress tolerance and reduced oxidative damage (Chen et al., 2017; Sheteiwy et al., 2021; Vishnupradeep et al., 2022).

Inoculation effects on proline and oxidative status are strain dependent. *Pseudomonas* have been reported to either increase or reduce proline accumulation under stress, whereas *Bacillus* often promotes proline-mediated osmoprotection (Patani et al., 2023; Chen et al., 2025). In this study, inoculated beans exhibited lower proline and MDA contents together with higher RWC, suggesting that bacterial inoculation mitigated drought-induced oxidative and osmotic stress. While proline accumulation contributes to osmotic adjustment and protection against oxidative damage, elevated proline levels can also reflect greater stress intensity (Silvente et al., 2012; Arteaga et al., 2020). Thus, the reduced proline concentration observed in inoculated plants, together with their improved physiological performance, is consistent with a lower demand for stress-induced osmotic adjustment rather than reduced drought tolerance.

Expression analysis of six drought-responsive genes in leaves sampled two weeks after drought recovery provided insight into the molecular mechanisms underlying the physiological responses observed in inoculated plants. The selected genes represent three major molecular components of drought adaptation: stress signaling (NCED3), osmotic adjustment through proline metabolism (P5CS10), and cellular protection mechanisms involving membrane stabilization, dehydration tolerance, and protein protection (LTP41, ERD10, LEA18, and DNAJ3). NCED3, a key regulator of ABA biosynthesis and drought signaling in common bean (López et al., 2020; Lee et al., 2021), was downregulated following inoculation, suggesting reduced activation of ABA-related stress responses after improved plant water status. Since ABA concentrations were not directly measured, this interpretation remains indirect. This response agrees with the partial restoration of gas exchange and higher RWC observed in treated plants, suggesting that treated plants experienced lower stress perception and therefore reduced activation of ABA-associated signaling pathways after recovery (El-Esawi et al., 2018). Similarly, LEA18 and ERD10, which encode dehydration-responsive proteins involved in cellular stabilization (Abid et al., 2017; Wu et al., 2023), showed reduced expression, consistent with reduced activation of dehydration-responsive pathways during recovery. Aligning with similar response reported in Pseudomonas-treated Arabidopsis under stress conditions (Marzorati et al., 2023). LTP41 remained elevated in non-inoculated plants after recovery, consistent with its role in membrane repair and cuticle reinforcement under drought, whereas its lower expression in treated plants aligns with reduced MDA accumulation and suggests limited membrane damage (Dong et al., 2022; Zhang et al., 2022).

Genes associated with protein protection and osmotic adjustment followed similar patterns. DNAJ3, a heat shock co-chaperone involved in protein folding and stress protection, is commonly induced under drought conditions (Hernández-Lucero et al., 2014; Wang et al., 2014; Dong et al., 2022); its downregulation in inoculated plants may indicate reduced activation of pathways associated with protein protection during recovery. Likewise, P5CS10, a key enzyme in proline biosynthesis frequently upregulated in drought-sensitive genotypes (Chen et al., 2010; López et al., 2020), showed reduced expression in treated plants, consistent with the lower proline concentrations measured in leaves and indicating diminished need for osmotic adjustment. Together, these transcriptional responses suggest that bacterial inoculation promoted a recovery-associated state characterized by reduced activation of drought-responsive pathways rather than prolonged stress responses.

Taken together, the *in vitro* and greenhouse experiments identified strain-specific contributions to drought stress mitigation and plant physiological recovery, providing the rationale for selecting candidate strains for preliminary field evaluation. Accordingly, BS-114 was selected as the core strain based on its strong improvement of photosynthetic performance under greenhouse drought conditions, while BSM-119 and BS-120 were included for their complementary stress-resilience traits, including high SOD activity, tolerance to abiotic stress and agriculturally relevant genomic features identified during previous characterization (Kaddoura et al., 2026). The three strains were selected within the *Bacillus* genus due to their spore-forming ability, which may improve bacterial persistence under fluctuating field conditions. The greenhouse study further indicated that combining complementary strains could enhance plant responses, supporting the development of a CONS rather than relying on a single isolate. Importantly, the field trial was not designed as a drought validation experiment, but rather as a preliminary trial of whether a stress-resilient bacterial consortium selected from controlled drought studies could maintain plant growth-promoting performance under open-field conditions.

Field evaluation in beans showed that CONS achieved crop productivity comparable to NPK fertilization and CB under open-field conditions. CB was included as a commercial control because algae-derived biostimulants are commonly used in agriculture as plant growth promoters, with reported benefits related to improved nutrient use, growth regulation, and stress adaptation through their naturally occurring bioactive compounds (Chabili et al., 2024). The comparable performance of CONS with both grower-standard fertilization NPK and CB suggests that the selected consortium has potential to support crop productivity under open-field conditions, extending their growth-promoting responses under controlled greenhouse experiments to a complex field environment. However, given the preliminary nature of these trials and the lack of biological replication in the tomato assessment, the observed yield differences should be interpreted cautiously and considered indicative trends rather than statistically validated treatment effects. Future multi-season replicated field evaluations under defined water-limiting scenarios will be required to confirm the consistency of CONS performance and to further understand its interactions with environmental factors and crop management practices.

## 5 Conclusions

This study evaluated the potential of endophytic *Bacillus* and *Pseudomonas* strains previously characterized for plant growth-promoting traits, including EPS, IAA, siderophore, organic acid, SOD, and proline production, as bioinoculants to improve crop performance under drought stress. The magnitude of the response was host- and strain-dependent. *In vitro*, bacterial inoculation produced more pronounced improvements in drought-related germination and seedling traits in common bean than in tomato, highlighting the importance of host–strain interactions during early plant development. Under greenhouse drought conditions, inoculated bean plants showed improved growth, water status, photosynthetic performance, reduced oxidative stress, and lower expression of drought-responsive genes following recovery. Individual strains were associated with distinct physiological responses, suggesting complementary functions that provided the rationale for developing a bacterial consortium. Consistent with these findings, the consortium delivered the strongest improvements in plant performance under controlled drought conditions and maintained promising effects under non-controlled agricultural conditions, where crop productivity was comparable to that achieved with standard fertilization and a commercial biostimulant. Together, these findings support the potential of functionally complementary endophytic bacterial consortia as microbial biostimulants for sustainable crop production. Future studies integrating multi-omic approaches with replicated multi-year field trials will be important to better understand the mechanisms underlying these plant–microbe interactions, evaluate their performance across different crops, and support the development of resilient agricultural systems under drought.

## Data Availability

The datasets presented in this study can be found in online repositories. The names of the repository/repositories and accession number(s) can be found in the article/**Supplementary Material**.
