# Supplementary material for: The functional roles of endophytic Bacillus and Pseudomonas bacteria to improve tomato and bean plants’ growth under induced-drought stress conditions
Source: Front Plant Sci. 2026 Sep 10;17:1905932. doi: 10.3389/fpls.2026.1905932 (PMC13600858; doi:10.3389/fpls.2026.1905932)
Supplement: Supplementary file 1 [file Table1.docx]

Supplementary Material

**Supplementary Table 1.** Primer sequences of genes used for qRT-PCR

| Gene name | Gene ID | Secuence (5` → 3´) (Forward/Reverse) | References |
| --- | --- | --- | --- |
| 9-cis-Epoxycarotenoid Dioxygenase | NCED3 | TTAAGGACCTGGCTTCCACATC | (1) |
|  |  | GTGATGGTGTTGGTGTTGGAAG |  |
| Lipid Transfer Protein | LTP41 | GCCTGAGCTACCTAAGAACCG | (2) |
|  |  | AACCCTGGGCTAACGCAC |  |
| Early Responsive to Dehydration | ERD10 | AGTATGAGACCGCAGAGAG | (3, 4) |
|  |  | AGGCTGTGCTTCTTCTCTC |  |
| Late Embryogenesis Abundant protein | LEA18 | AGTAAACCTTGAAGGGCTTCC | (4, 5) |
|  |  | AGTGGCTGGGGACTTAGAG |  |
| Heat Shock Protein DnaJ | DnaJ3 | ATGTCATATGCTCCAAGTGCAC | (4, 6) |
|  |  | GGCAGCGATCTCTATCATTG |  |
| Pyrroline-5-Carboxylate Synthase | P5CS10 | GATGTTGAGGGCCTTTACAGTG | (1) |
|  |  | CTGCCCAATCTTGACTTGTCTC |  |
| actin-11 | ACT11 | TGCATACGTTGGTGATGAGG | (7) |
|  |  | AGCCTTGGGGTTAAGAGGAG |  |

*Supplementary Figure 1: (A) Relative water content (RWC) for well-watered treatments (w+). Values are means ± SD (n = 5). Different letters indicate significant differences among treatments (two-way ANOVA followed by Tukey’s test, P < 0.05).*

***References***

1.	López CM, Pineda M, Alamillo JM. Differential Regulation of Drought Responses in Two Phaseolus vulgaris Genotypes. Plants. 2020;9(12):1815.

2.	Dong X, Zhu H, Hao X, Wang Y, Ma X, Zhao J, et al. Genome-Wide Identification of Common Bean PvLTP Family Genes and Expression Profiling Analysis in Response to Drought Stress. Genes. 2022;13(12):2394.

3.	Wu G, Tian N, She F, Cao A, Wu W, Zheng S, et al. Characteristics analysis of Early Responsive to Dehydration genes in Arabidopsis thaliana (AtERD). Plant Signaling & Behavior. 2023;18(1):2105021.

4.	Hernández-Lucero E, Rodríguez-Hernández AA, Ortega-Amaro MA, Jiménez-Bremont JF. Differential Expression of Genes for Tolerance to Salt Stress in Common Bean (Phaseolus vulgaris L.). Plant Molecular Biology Reporter. 2014;32(2):318-27.

5.	Abid G, M’hamdi M, Mingeot D, Aouida M, Aroua I, Muhovski Y, et al. Effect of drought stress on chlorophyll fluorescence, antioxidant enzyme activities and gene expression patterns in faba bean (Vicia faba L.). Archives of Agronomy and Soil Science. 2017;63(4):536-52.

6.	Dong A, Zenda T, Liu X, Wang Y, Li J, Yang Y, et al. Transcriptome analysis of drought tolerance and utility assessment of DnaJ gene expression as a potential index for drought resistance evaluation in maize. Euphytica. 2022;218(9):136.

7.	Borges A, Tsai SM, Caldas DGG. Validation of reference genes for RT-qPCR normalization in common bean during biotic and abiotic stresses. Plant Cell Reports. 2012;31(5):827-38.
